# Reduced penetrance *BRCA1* and *BRCA2* pathogenic variants in clinical germline genetic testing

**DOI:** 10.1038/s41698-024-00741-4

**Published:** 2024-11-02

**Authors:** Tuya Pal, Erin Mundt, Marcy E. Richardson, Elizabeth Chao, Tina Pesaran, Thomas P. Slavin, Fergus J. Couch, Alvaro N. A. Monteiro

**Affiliations:** 1 Department of Medicine, Vanderbilt-Ingram Cancer Center, University Medical Center, Vanderbilt University, Nashville, TN USA; 2 Myriad Genetics, Salt Lake City, UT USA; 3 Ambry Genetics, Aliso Viejo, CA USA; 4 Department of Laboratory Medicine, Mayo Clinic, Rochester, MN 55905 USA; 5 Department of Cancer Epidemiology, H. Lee Moffitt Cancer Center and Research Institute, Tampa, FL 33612 USA

**Keywords:** Breast cancer, Cancer genetics

## Abstract

Prior studies have suggested the existence of reduced penetrance pathogenic variants (RPPVs) in *BRCA1* and *BRCA2* (*BRCA*) which pose challenges for patient counseling and care. Here, we sought to establish RPPVs as a new category of variants. Candidate *BRCA* RPPVs provided by two large clinical diagnostic laboratories were compiled to identify those with the highest likelihood of being a RPPV, based on concordant interpretations. Sixteen concordant candidate *BRCA* RPPVs across both laboratories were systematically assessed. RPPVs included missense, splice site, and frameshift variants. Our study establishes RPPVs as a new class of variants imparting a moderately increased risk of breast cancer, which impacts risk-informed cancer prevention strategies, and provides a framework to standardize interpretation and reporting of *BRCA* RPPVs. Further work to define clinically meaningful risk thresholds and categories for reporting *BRCA* RPPVs is needed to personalize cancer risks in conjunction with other risk factors.

## Introduction

Approximately 5–10% of breast cancers (BC) are attributable to pathogenic/likely pathogenic variants (PVs) in high penetrance BC genes, with an additional 20% attributed to moderate and low penetrance genes^1–8^. Cancer risks associated with PVs in high penetrance genes may be defined as a relative risk (RR) > 4-fold, and may be as high as 10–20 fold^9^. For example, female *BRCA* carriers typically have a 60–70% lifetime BC risk compared to 12% in the general population^1^. Ovarian cancer lifetime risk for *BRCA1* and *BRCA2* carriers is up to 44% and 17%, respectively, compared to 1.5% in the general population^9^.

The National Comprehensive Cancer Network (NCCN) recommended care for carriers of PVs differs between high and moderate penetrance genes^10^. While high-risk screening through annual breast MRI and mammograms is appropriate for females with PVs in both high- and moderate- penetrance genes, risk-reducing mastectomy (RRM) is only included as an option for those with PVs in high penetrance genes^10^. Notably, identification of these PVs is now considered in therapy selection with the FDA approval of PARP1 inhibitors for breast, ovarian, pancreatic, and prostate cancers^10–15^.

While current clinical classification of variants is binary (pathogenic or benign), the degree of risk, a continuous variable which is influenced by many factors including variant type, specific alteration, genetic background, behavioral, and environmental factors is not factored into clinical classification. In the classic high penetrance *BRCA* genes, prior studies have suggested the existence of specific PVs that impart a reduced risk of cancer compared with high-penetrance protein truncating variants^16–19^.

Despite prior data to support the existence of reduced penetrance pathogenic variants (RPPVs), there remains a lack of standard nomenclature for interpreting and reporting these variants, resulting in varying terminology such as “intermediate risk”, “hypomorphic”, “reduced risk”, “atypical risk”, and “special interpretation”, among others. These variants are both a challenge to identify due to discordant classifications, as well as to manage clinically due to uncertainty around clinical recommendations. The lack of harmonization is likely due to variant classification models that are designed for typical risk Mendelian variants, which rely on the afore-mentioned binary classification^20,21^. These models fall short in incorporating the magnitude of risk. Consequently, the variations in risk and the lack of clarity regarding terminology have resulted in inconsistent interpretation and reporting of these variants which account for many of the ‘conflicting interpretations’ across labs reflected in ClinVar^22^.

Given that risk is a continuum and evidence that variants with atypical penetrance exist, we hypothesized that clinically-relevant *BRCA* RPPVs exist. To address this need, we sought to identify and characterize these variants to support RPPVs as a new variant classification category. Specifically, we compiled and systematically assessed candidate *BRCA* RPPVs reported by two large US-based commercial clinical diagnostic laboratories based on concordant interpretations. We propose to establish *BRCA* RPPVs as a new class of PVs that are clinically relevant to better guide preventive and clinical management decisions for carriers.

## Methods

The clinical history and genomic instability score analyses used in this manuscript was performed using de-identified data obtained during the course of routine healthcare operations. Only aggregate data are presented in the manuscript. WGC IRB (formerly Western Institutional Review Board) determined the study to be exempt from the Office for Human Research Protections Regulations for the Protection of Human Subjects.

### Selection of variants

Sets of candidate RPPVs in *BRCA1* (OMIM *113705) and *BRCA2* (OMIM * 600185) provided by Myriad Genetics and Ambry Genetics were compared for concordant interpretations. Because individual laboratories have different but complementary approaches in identifying potential RPPVs, we hypothesized that variants present in both datasets are likely to constitute those with the highest prior likelihood of being a true RPPV.

Initial identification of candidate RPPVs was based on several approaches including 1) identification of biallelic Fanconi Anemia-affected carriers; 2) consideration of variant type where splicing, missense, and nonsense-mediated RNA decay (NMD)-escaping variants which are more likely to be partially functional than truncating variants; 3) evaluation of laboratory-validated cancer history weighting models (HWAs); 4) incorporation of published variant-specific risk estimation data; 5) extrapolation of a reduced-penetrance interpretation onto close match variants that are expected to have the same effect; and 6) inclusion of functional data. The final set of 16 variants was then systematically assessed for pathogenicity and evidence of reduced penetrance.

### Fanconi anemia (FA)

Identification of biallelic FA -affected carriers was used as evidence of reduced penetrance. This approach is based on evidence that *Brca1-*null and *Brca2*-null mice are embryonic lethal^23–27^ and no homozygous or compound heterozygous carriers of high-frequency founder pathogenic variants (*BRCA1* c.68_69delAG, *BRCA1* c.5266dupC, and *BRCA2* c.5946delT) have been observed suggesting that two alleles with complete loss-of-function alterations are also embryonic lethal in humans. On the other hand, homozygous or compound heterozygous carriers of reduced (partial loss) function variants develop FA^28,29^. A carrier affected with FA is a line of evidence towards pathogenicity of the variant under the PM3 code in the current American College of Medical Genetics/Association of Molecular Pathologists (ACMG/AMP) model of variant interpretation for both alterations identified in a biallelic patient^20^. Under the evidence-based assumption that at least one of the two Likely Pathogenic/Pathogenic (LP/P) alleles in a FA-affected carrier must be attenuated, one can infer the identity of the *BRCA* RPPV by evaluating several factors including: 1) homozygosity (versus. compound heterozygosity) in an affected carrier; 2) recurrence of one alteration in multiple affected patients with different LP/P variants in trans (i.e., in different homologous chromosomes); 3) variant type favoring missense, splicing, or NMD-escaping variants as most likely to have reduced penetrance due to plausible, partially retained function.

### Homologous recombination deficiency (HRD) score

The MyChoice® CDx Myriad HRD Companion Diagnostic test examines ovarian cancer tumors using both *BRCA1/BRCA2* pathogenic variant status and genomic instability. Previous studies have shown that a high genomic instability score (GIS) is observed in 95% of tumors with a pathogenic *BRCA1* or *BRCA2* variant^30^.

### Personal and family cancer history

Family history models are internally developed and validated cancer history weighting algorithms (HWAs) and were used to evaluate personal and family cancer histories of variant carriers^31,32^. These models compare the histories of individuals with a variant of interest to matched cases (carriers of known pathogenic variants in the gene) and controls (individuals with no known pathogenic variants) in the cohort of each respective laboratory. The number of probands for each individual variant used in the HWAs is listed in Supplementary Table 1.

### Integration of functional data

Specific instances of validated functional assays (>80% sensitivity and >80% specificity) performed in any published work were collated for all missense variants in this study (*BRCA1* p.(Arg1699Gln); *BRCA2* p.(Trp2626Cys) and p.(Leu3101Arg)). For functional data integration we harmonized data by following the reported conclusion of whether a given variant had a significant impact on the function being assayed, and transformed these results into a binary categorical result (impact versus no impact) as described previously^33^. Data for variants described here were obtained from published work^34–41^. Briefly, we assigned “0” to a variant that did not lead to a significant impact on the function (functionally normal); and “1” to a variant that was functionally abnormal. Scores “0” and “1” are presumably associated with (likely) benign and (likely) pathogenic variants, respectively. To obtain final calls, we applied the ACMG/AMP criteria applicable to functional assays, which denote supporting (BS3_supporting, PS3_supporting), moderate (BS3_moderate, PS3_moderate), and strong (BS3, PS3) evidence against and for pathogenicity, respectively, from well-established in vitro or in vivo functional studies^20,42,43^.

## Results

**Fig. 1:**
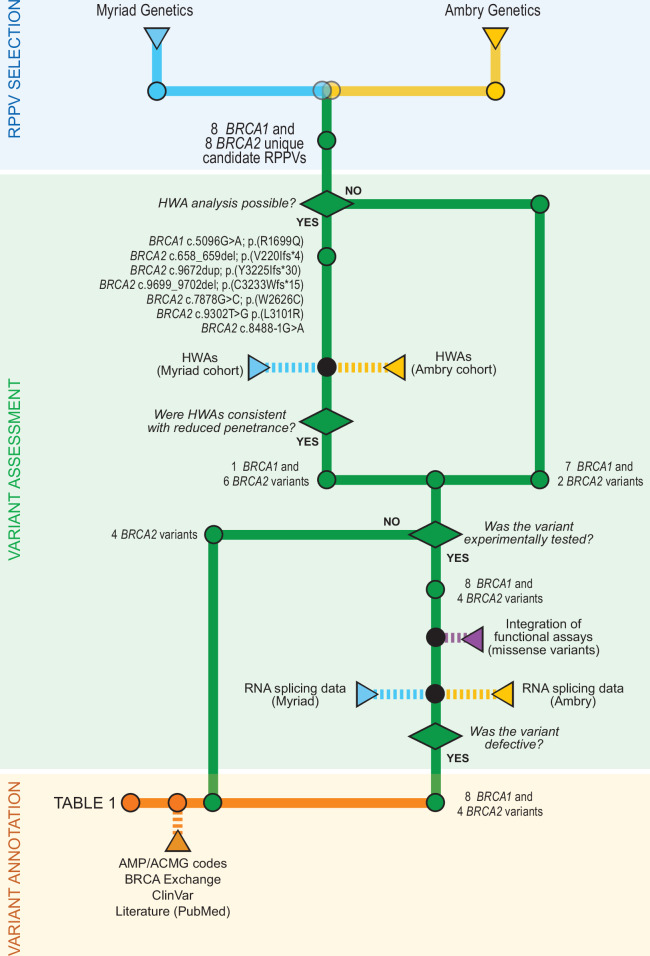
Subway chart outlining the study design. HWA, cancer family and personal history weighing algorithms; RPPV, reduced penetrance pathogenic variant.

Each laboratory weighted the evidence according to their own internal rules, procedures, and guidelines to select candidate RPPVs (Fig. 1). Sixteen variants were present in both sets and thus were considered to have sufficient prior likelihood of being a RPPV (Table 1).

**Table 1:** List of sixteen consensus *BRCA1* and *BRCA2* reduced penetrance pathogenic variants (RPPVs)

RPPV^a^	Variant Type	FA patients	HWA score (both labs)	Functional data (ACMG evidence criteria)^b^	ClinVar^c^	Variant Level risk
*BRCA1* c.5096 G > A; p.(Arg1699Gln)	Missense	No	Not high risk	Yes (PS3)	P^***^	RR 2-4 (Refs. ^19,44^)
*BRCA1* c.671-1delins6	Splice	No	ND^d^	Alternative in-frame isoforms with uncertain functional impact (RNA) Refs. ^46–48^, Myriad, Ambry internal data		ND
*BRCA1* c.671-2 A > T	Splice	No	ND	*Ibid*.	P^**^	ND
*BRCA1* c.671-2 A > G	Splice	No	ND	*Ibid*.	Conflict (LP/P/VUS)*	ND
*BRCA1* c.671-2 A > C	Splice	No	ND	*Ibid*.	LP/P**	ND
*BRCA1* c.671-1 G > T	Splice	No	ND	*Ibid*.	Conflict (LP/P/VUS)*	ND
*BRCA1* c.671-1 G > C	Splice	No	ND	*Ibid*.	Conflict (LP/VUS)*	ND
*BRCA1* c.671-1 G > A	Splice	No	ND	*Ibid*.	P**	ND
*BRCA2* c.658_659del; p.(Val220Ilefs*4)	Frameshift	Recurrent (Refs. ^50–53,72,73^)	Not high risk	ND	P***	ND
*BRCA2* c.9672dup; p.(Tyr3225Ilefs*30)	Frameshift	Recurrent (Refs. ^28,55,56^)	Not high risk	ND; NMD-escaping	P***	ND
*BRCA2* c.9699_9702del; p.(Cys3233Trpfs*15)	Frameshift	Recurrent (Ref. ^54^., Ambry internal data)	Not high risk	ND; NMD escaping	LP/P***	ND
*BRCA2* c.7878 G > C; p.(Trp2626Cys)	Missense	Recurrent (Refs. ^57,58,74,75^)	Not high risk	Yes (PS3)	P***	ND
*BRCA2* c.7878 G > T; p.(Trp2626Cys)	Missense	No	ND	Yes (PS3)	P*	ND
*BRCA2* c.9302 T > G; p.(Leu3101Arg)	Missense	Yes (Ambry personal communication)	Not high risk	Incomplete aberrant splicing, some in frame effects (Refs. ^76–78^)	Conflict (LP/P/VUS)*	ND
*BRCA2* c.8488-1 G > A	Splice	Yes, homozygous (Ref^28^)	Not high risk	Yes (PS3)	P***	ND
*BRCA2* c.8488-1 G > T	Splice	Yes (Myriad)	ND	ND	P**	ND

^**a**^Reduced penetrance pathogenic variant (RPPV) in HGVS designation.

^**b**^See Fig. 3.

^c^ND, not determined. ^d^ClinVar interpretations (*P* pathogenic, *LP* likely pathogenic, *VUS* variant of uncertain significance); ClinVar review status, asterisks represent gold stars; ***, reviewed by expert panel; **, criteria provided, multiple submitters, no conflicts; *, criteria provided, conflicting interpretations (or single submitter).

### BRCA1 RPPVs

**Fig. 2:**
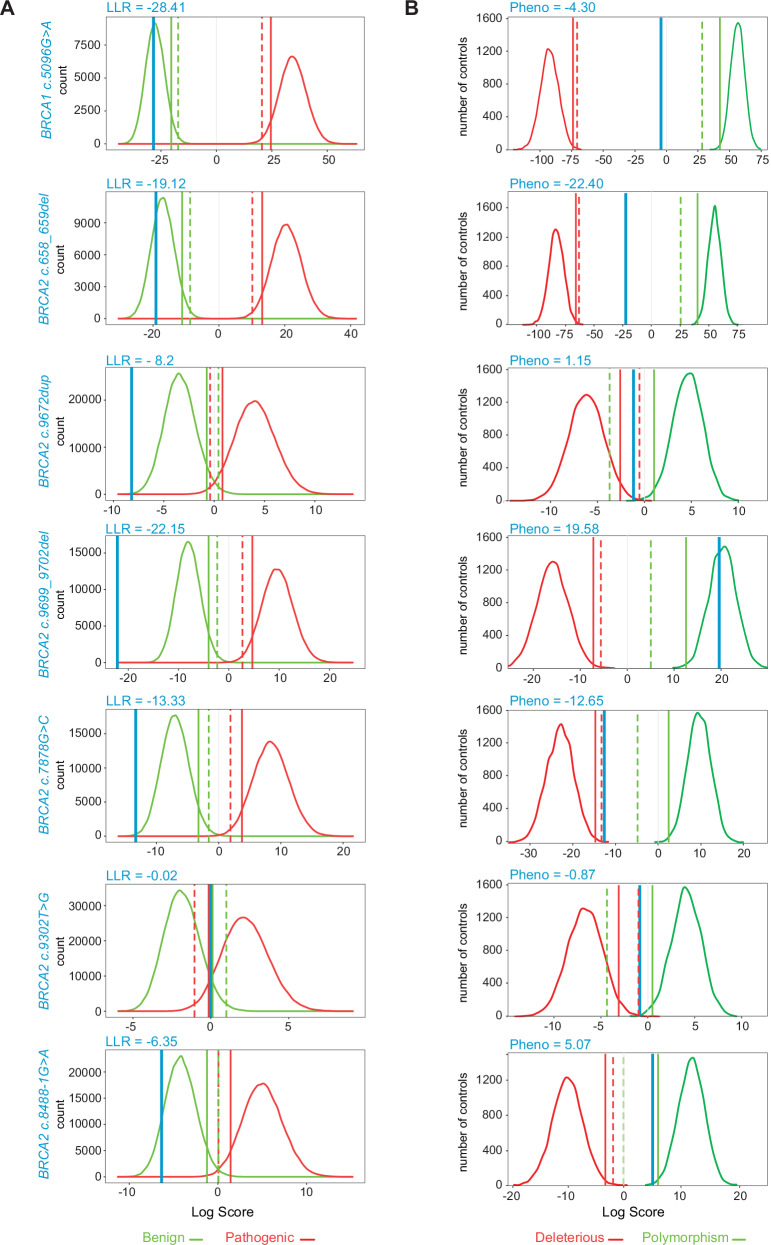
Clinical history curves for seven RPPVs. Aggregate clinical data of variant carriers (blue line) is plotted relative to the distribution of carriers of known pathogenic (red curves) and known benign (green curves). **A** History Weighing Algorithm (HWA; Ambry Genetics)^31^ assessment of aggregate family history for carriers of variants indicated (blue vertical line) plotted relative to the distribution of simulated control groups for benign (green curve) and pathogenic (red curve) variants. Dashed and solid vertical lines represent the conservative 95th and 99th percentile confidence bounds for pathogenic and benign curves for the control groups. Each count refers to one simulated dataset with the same number of probands as the test dataset. Each benign and pathogenic curve is comprised of 100,000 simulated datasets with the same number of probands as the test dataset and count refers to the number of those simulated datasets. LLR, Log Likelihood Ratio. **B** History Weighing Algorithm (HWA; Myriad Genetics)^32^ assessment of aggregate family history for carriers of variants indicated (dark blue vertical line) plotted relative to the distribution of simulated control groups for benign (green curve) and pathogenic (red curve) variants.

**Fig. 3:**
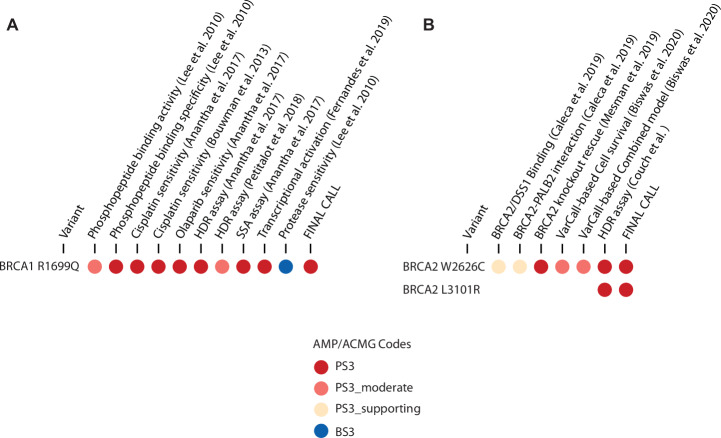
Functional evidence for pathogenicity for missense RPPVs. Available published and unpublished cumulative functional evidence for three RPPVs in *BRCA1* (**A**) and *BRCA2* (**B**) were compiled and coded per ACMG-AMP codes (PS3 functional evidence towards pathogenic; BS3 functional evidence towards benign) and strength (down-weighted from baseline ‘strong’ to either ‘moderate’ or ‘supporting’ where denoted)^33,79^. A final call on functional strength is provided based on the cumulative functional evidence.

For *BRCA1*, there were eight overlapping candidate RPPVs that had been observed in patients. They included one missense and seven splicing site variants. The only missense variant in this set, *BRCA1* c.5096 G > A (p.(Arg1699Gln)), had family histories inconsistent with a high penetrance *BRCA1* variant according to HWAs from both laboratories (Fig. 2). Analysis of previously published functional data for 11 different validated assays show that 10/11 scored c.5096 G > A (p.(Arg1699Gln) as PS3 or PS3_moderate^33^. Overall, the functional evidence indicates that this variant is defective across a wide range of assays (Fig. 3). Through modified segregation analysis of 30 families, this variant was shown to have reduced penetrance, compared with the average penetrance truncating *BRCA1* mutation (*p* = 0.0002), with estimated cumulative risks to age 70 of breast or ovarian cancer of 24%^19^. This was confirmed in larger cohorts of families ascertained internationally by ENIGMA (Evidence-based Network for the Interpretation of Germline Mutant Alleles) consortium members^16,44^. The cumulative risk of BC and ovarian cancer by age 70 years was 20% and 6%, respectively^44^. Taken together, the evidence supports the pathogenicity and reduced penetrance of this variant justifying the designation of c.5096 G > A (p.Arg1699Gln) as an RPPV.

Interestingly, all the other seven candidate *BRCA1* RPPVs impact the canonical c.671 splice acceptor site in intron 10 (intron 8 for coding exon nomenclature). They were considered RPPV-*BRCA* based on known alternative splicing in this exon. Alterations at this acceptor site cause aberrant splicing involving the skipping of all or part of exon 11 with predicted loss of nearly 60% of the BRCA1 protein^45^. These isoforms, however, are in frame and are found as naturally occurring, in-frame events. Exon 11 skipping (r.671_4096del) is a naturally occurring minor event in peripheral blood leukocytes while exon 9_11 skipping (r.548_4096del) is a predominant isoform found in whole blood leukocytes, lymphoblastoid cell lines and non-malignant breast cells. An additional isoform causing in-frame skipping of the N-terminal part of Exon 11 (r.788_4096del) is a predominant isoform in lymphoblastoid cell lines, non-malignant breast cells, and BC cell lines^46^. Although each of these naturally occurring, in frame events result in the loss of a large part of the protein, the impacted region is not in a recognized functional domain and in vitro and mouse studies suggest a partial effect with impaired cellular localization, reduced DNA damage repair and a longer embryonic survival than *Brca1*-null embryos (Table 1)^47–49^.

### BRCA2 RPPVs

Overlapping RPPV-*BRCA2* variants included three frameshift variants, *BRCA2* c.658_659delGT, c.9672dupA, and c.9699_9702delTATG. The latter two are C-terminal, NMD-escaping frameshifts which may retain partial function. Each of these variants were identified in at least one FA-affected patient^28,50–56^ and HWAs from both laboratories suggest that they are not high penetrance *BRCA2* variants (Fig. 2). The *BRCA2* c.658_659delGT variant is expected to undergo NMD and the mechanism whereby it may have reduced function is not understood, however, it is recurrent in FA-affected individuals.

There were also two spliceogenic alterations of the same nucleotide on in the *BRCA2* RPPV candidate lists from both laboratories. Variant c.8488-1 G > A which, despite being a canonical splice site alteration, leads to an incomplete splicing effect and does not behave like a high penetrance *BRCA2* truncating variant on HWA (Fig. 2). Variant c.8488-1 G > T is inferred to be a RPPV based on a similar predicted splice effect as the c.8488-1 G > A.

Lastly, there were two *BRCA2* missense variants that overlapped each laboratories’ lists, both of which lacked a HWA score suggestive of a highly penetrant variant and each of which were present in FA patients: c.7878 G > C (p.(Trp2626Cys))^57,58^ and c.9302 T > G (p.(Leu3101Arg)) (Ambry internal data) (Table 1). Of note, the *BRCA2* RPPV interpretation is extrapolated onto a distinct nucleotide variant at the same codon (c.7878 G > T) which results in the same amino acid substitution, p.(Trp2626Cys), as the c.7878 G > C. Analysis of published functional data for c.7878 G > C (p.(Trp2626Cys) shows that 6/6 validated assays score this variant as defective (PS3_supporting, PS3_moderate, and PS3). Overall, the evidence indicate that this variant is defective across a wide range of assays (Fig. 3). Results from only one validated assay (homologous-directed recombination) was available for c.9302 T > G (p.(Leu3101Arg)) and scores as defective (PS3) (Fig. 3).

### HRD scores

Analysis of HRD scores was not possible for individual variants due to their low frequency in the population. Collectively, three RPPVs described here (*BRCA1* c.5096 G > A (p.Arg1699Gln), *BRCA2* c.658_659del (p.Val220Ilefs*4), and *BRCA2* c.8488-1 G > A) were observed in 14 ovarian tumors and five of which (35.7%) had a low (score < 42) Genomic Instability Score (GIS) and nine of which (64.3%) had a high GIS (score ≥ 42). The percentage of ovarian tumors herein over the GIS threshold of 42 was lower than previously reported (~95%)^59^.

## Discussion

Findings from our study support RPPVs as a new class of PVs and provides a framework to standardize their interpretation and reporting. We propose that this clinically-relevant class include variants with a 2-4-fold risk of BC. Based on data compiled on 16 *BRCA* variants, we provide compelling evidence that these variants constitute susceptibility alleles with reduced penetrance when compared to complete loss of function (*e.g*., protein truncating) variants. Consequently, harmonized variant interpretations, vocabulary, standardized reporting, and care strategies will be critical to optimize risk-informed care.

Our findings highlight the differences and similarities in approaches taken by each of the laboratories in identifying *BRCA* RPPVs. Of note, the ACMG/AMP variant classification model is amongst the most widely used models to classify variants with Mendelian inheritance, however, there are acknowledged limitations in this model to identify and classify variants with atypical risks^20,60,61^. Due to a lack of consensus guidance on how to identify and interpret RPPVs, there is significant confusion and discordance in classifying variants with evidence for pathogenicity but atypical presentation or risk.

Carriers of *BRCA* RPPVs may benefit from more complex risk assessment models that incorporate risk-associated single nucleotide variants (SNVs) aggregated into polygenic risk scores (PRS). Although PRS have been shown to have a smaller contribution to risk modification in carriers of PV in *BRCA1* and *BRCA2* than in non-carriers, recent data indicates that modification of risk in other moderate-penetrance BC genes is similar to that observed in women without a PV^9,62,63^. This observation raises the possibility that risks conferred by RPPVs in highly penetrant genes may be significantly modified by PRS. Further studies will be necessary to determine the contribution of PRS to risk modification in carriers of *BRCA* RPPVs.

The small set of RPPVs in this study show that missense, NMD-escaping frameshift, and splicing alterations can lead to hypomorphism (partial loss of function) and reduced penetrance. Variant c.658_659del is a frameshift variant which leads to a premature termination codon (PTC) in exon 8. Therefore, it is expected to produce a severely truncated protein that is targeted by nonsense-mediated mRNA decay^64^. Nevertheless, it was found in Fanconi anemia patients suggesting that the allele produces a protein with residual function. At present, the mechanism underlying the variant’s function is unknown. Interestingly, several *BRCA2* alleles that lead to PTC have been shown to also produce transcripts that bypass the PTC by exon skipping that restore or maintain the original reading frame^65–67^. Functional studies, using cells from carriers may inform on whether exon skipping transcripts that exclude exons 7-8 are produced and whether deleted protein sequences are dispensable for BRCA2 function.

There are several clinical implications that stem from the identification of RPPVs. Currently, carriers of RPPVs are at risk of both over and undertreatment. For example, overtreatment may result when care providers offer risk-reducing mastectomy (RRM) to female RPPV carriers with or without a BC diagnosis, even though their risks may be similar to carriers of PVs in moderate penetrance genes such as *ATM* or *CHEK2* for which RRM is not recommended^68^. On the other hand, care providers may interpret the effects to be not as severe as truncating PVs and consider RPPVs as benign or variants of uncertain significance, and not offer high risk screening for BC. Notably, clinical recommendations for RPPV carriers with ovarian cancer may not differ from carriers of high penetrance *BRCA* PVs. This is because even with significantly reduced penetrance, RPPV-associated ovarian cancer risks would still likely exceed the current threshold of ~5% lifetime risk for risk-reducing salpingo oophorectomy consideration^10^.

Our findings are of broader relevance beyond *BRCA* variant classification, and there is robust evidence to support risk as a continuum in other hereditary BC genes in which specific variants deviate from typical gene-specific penetrance estimates. For example, among genes considered to be of moderate penetrance, such as *CHEK2* and *ATM*, there are specific variants identified in these genes that deviate from the 2–4-fold risk of BC typically imparted by PVs in these genes^69–71^.

We have shown that clinically meaningful RPPVs can be identified. Penetrance estimates derived from segregation analysis or large case-control studies will be important to further characterize RPPVs. Large international networks such as the Breast Cancer Association Consortium and the ENIGMA (Evidence-based network for the Interpretation of Germline Mutant Alleles) consortium will be instrumental to identify and include RPPV carriers to achieve statistical power. However, we recognize that it may not be possible to determine risk estimates associated with every individual RPPV due to the low number of carriers. Therefore, it will be imperative to identify predictors of reduced (as well as ‘atypical’) penetrance which may involve the lines of evidence used in the current study. In particular, we propose that once specific variants are benchmarked against a set of known RPPVs, a functional study may be able to predict intermediate function/reduced penetrance for other rare variants.

One of the limitations of our study is that the available functional integration^33^ did not account for intermediate scores. The ability of a functional assay to distinguish highly from moderately functionally impaired variants depends on its accuracy, threshold of pathogenicity (calibration), and dynamic range. Therefore, RPPVs are expected to score as intermediate in some assays, or alternatively to have discordant scores across different assays, reflecting differences in calibration and dynamic range. It will be critical to compare the RPPVs identified in this study with highly penetrant variant alleles in a comprehensive integration of functional data that accounts for intermediate results so that assays can be properly calibrated.

An important unanswered question is whether tumors arising in RPPV carriers behave differently than traditional (high penetrance) *BRCA* PVs regarding response to cancer therapy, in particularly PARP inhibitors. The sample size of tumors with a RPPV was limited, precluding ability to determine how these variants may impact genomic instability overall, measured through GIS. The percentage of high-GIS tumors harboring one of the RPPVs was not as high as seen with fully penetrant variants, however, many of these tumors had elevated HRD GISs suggesting those patients will still benefit from PARP inhibitor therapy. Of note, all three RPPV identified were seen in tumors with both high and low GIS.

This study highlights unmet clinical needs to establish this new category of RPPVs in the *BRCA* genes, through identifying, characterizing, and harmonizing reporting of these variants across labs, researchers, and public databases like ClinVar. We specifically identified 16 consensus RPPVs that are not as penetrant as traditional (i.e., truncating) *BRCA* PVs, which are estimated to impart up to a 20-fold increased risk for BC. The reduced risks associated with these variants provide the foundation upon which to develop aligned clinical recommendations. Through establishing and defining this new class of variants, refining risk categories, and integrating additional factors in BC risk assessment, we expect that the current care paradigm will shift to view risk on a continuum rather than one that is binary. The work herein will help realize the ultimate goal of providing personalized and individualized risk assessment and care.

## Supplementary information

Supplementary Table 1

## Data Availability

Data supporting the history weighting algorithm and genomic instability findings of this study are available from Myriad Genetics and Ambry Genetics by request with reasonable accommodations to protect patient privacy and safety concerns, and that the data will not be made open access. Ambry Genetic and Myriad Genetics supports open data sharing. Functional data is derived from previously published work. Integrated and harmonized functional scores for individual variant are available upon request. Requests can be initiated by contacting the corresponding authors by email.

## Code availability

No custom code was generated for this manuscript. Codes used to generate family history scores are tailored to the internal structure of data and are, therefore, not provided. Methods for the HWA models can be found in earlier publications^31,32^.
