# Supplementary material for: Reduced penetrance BRCA1 and BRCA2 pathogenic variants in clinical germline genetic testing
Source: NPJ Precis Oncol. 2024 Nov 2;8:247. doi: 10.1038/s41698-024-00741-4 (PMC11531542; doi:10.1038/s41698-024-00741-4)
Supplement: Supplementary file 1 — Supplementary Table 1 [file 41698_2024_741_MOESM1_ESM.pdf]

Supplementary Table 1: Number of probands used in each HWA

Variant	Ambry	Myriad
c.8488-1G>A	52	151
c.7878G>C (p.Trp2626Cys)	87	222
c.658_659del (p.Val220Ilefs*4)	213	956
c.5096G>A (p.Arg1699Gln)	533	417
c.9672dupA (p.Tyr3225Ilefs*30)	43	73
c.9699_9702del (p.Cys3233Trpfs*15)	100	266
c.9302T>G (p.Leu3101Arg)	24	75
